# Gap Measurements in Aerospace Engineering

**DOI:** 10.3390/s25103059

**Published:** 2025-05-12

**Authors:** Xinyuan Zhao, Chao Zhang, Long Xu, Tao Wang, Pei Li, Heng Zhang, Jun Yang

**Affiliations:** 1 Chongqing Jiaotong University, Chongqing 400074, China; 2 Chongqing Institute of Green and Intelligent Technology, Chinese Academy of Sciences, Chongqing 400714, China; 3 Chongqing School, University of Chinese Academy of Sciences, Chongqing 400714, China; 4 Chengdu Aircraft Industrial (Group) Co., Ltd., Chengdu 610073, China

**Keywords:** aerospace engineering, micro-gap measurement, high-precision sensors, measurement methods, dynamic measurement

## Abstract

Advanced precision gap measurement technologies play a pivotal role in ensuring the design and operational efficiency of aerospace systems. Gaps between aircraft components directly influence assembly accuracy, performance, and safety. This review comprehensively explores the state-of-the-art in precision gap measurement technologies used in the aerospace sector. It categorizes and analyzes various sensors based on their operating principles, including optical, electrical, and other emerging technologies. Each sensor’s principle of operation, key advantages, and limitations are detailed. Furthermore, the paper identifies the significant challenges faced in aerospace gap measurement and discusses future development directions, emphasizing the need for enhanced accuracy, adaptability, and resilience to environmental factors. This study provides valuable insights for researchers and engineers in the field, guiding future innovations in precision gap measurement technologies to meet the evolving demands of aerospace manufacturing and maintenance.

## 1. Introduction

With the continuous advancement of aerospace technology, particularly in supersonic flight and space exploration [1,2,3,4], the control requirements for micro-gaps in aircraft manufacturing and assembly processes are becoming increasingly stringent. From the fuselage frame to the wings and tail section, countless precise joining operations are required to ensure the safe operation of the aircraft and maintain optimal aerodynamic performance. Especially when the aircraft operates under extreme conditions such as high speeds, high pressures, extreme temperatures, and intense vibrations [5,6], even minor assembly deviations can introduce significant safety risks, potentially leading to structural failures or catastrophic incidents [7]. Furthermore, excessive micro-gaps may cause structural loosening, stress concentration, and material fatigue, while insufficient micro-gaps can result in interference, component damage, or system malfunctions. More specifically, improper clearances between aircraft engine blade tips can compromise operational safety, leading to efficiency losses and increased fuel consumption [8]. Similarly, unintended micro-gaps in critical structural areas, such as those between aircraft skins, can disrupt aerodynamics and degrade overall performance [9]. Research [10] has pointed out that even millimeter-scale discrepancies may have far-reaching consequences for flight stability and safety. Table 1 outlines the assembly gap requirements for various components. Specifically, the gap for the high-pressure turbine blade tips ranges from 0.199 to 0.625 mm [11]; for aircraft skin assembly, it is between 0.15 and 0.4 mm [12,13]; door stop block assembly requires a gap of 0 to 0.5 mm [14]; for the wing component assembly, the gap should be between 1 and 1.5 mm [9,15,16]; and the gap for turbine blade assembly is between 0.2 and 0.3 mm [17,18]. Therefore, accurate micro-gap measurement is not only of theoretical importance but also of significant engineering value in achieving high-quality manufacturing, extending aircraft service life, and ensuring flight safety.

**Figure 1:**
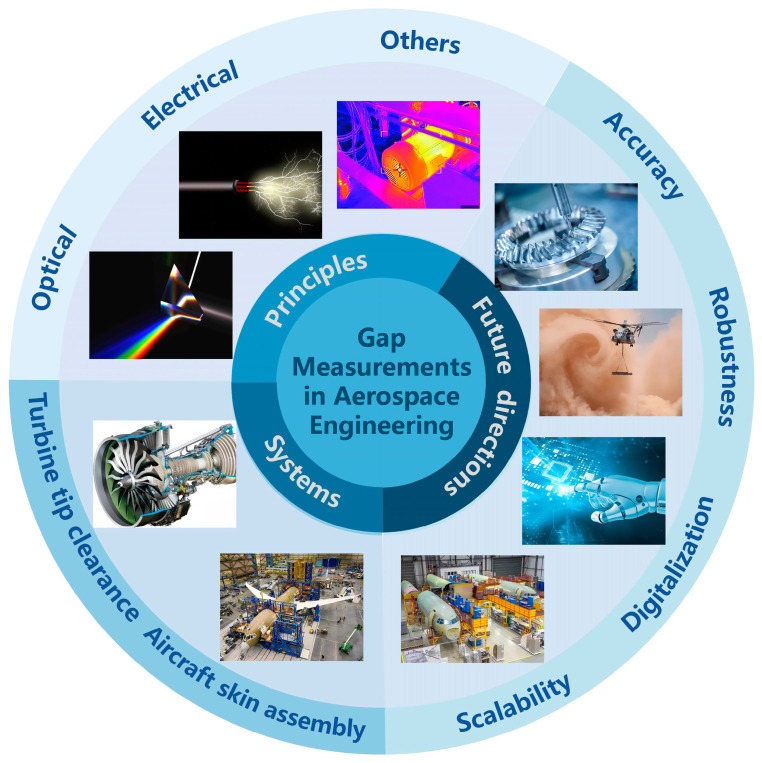
Measurement principles, systems, and development directions of micro-gap measurement sensors.

This review provides an in-depth analysis of precision micro-gap measurement sensor technologies within the aerospace sector. The overall framework of this study is shown in Figure 1. It begins with an examination of the fundamental measurement principles, followed by a comparison of the advantages and limitations of the commonly used sensor technologies. Furthermore, this review highlights application systems for micro-gap measurements and explores potential directions for future development. Special attention is given to the performance of these sensors in complex aerospace environments, particularly addressing the challenges of adaptability, accuracy, and reliability under extreme operating conditions.

## 2. Micro-Gap Measurement Sensors and Principles

Accurate micro-gap measurement is paramount in aerospace applications, as even minute discrepancies can significantly impact the performance, safety, and longevity of aircraft. To address this challenge, a variety of precision measurement sensors have been developed, each based on distinct operating principles. These sensors are designed to meet the specific needs of different aerospace applications, facilitating efficient design, manufacturing, and maintenance processes.

This section provides an overview of the commonly used precision micro-gap measurement sensors in the aerospace industry. We will classify these sensors according to their underlying measurement principles: optical, electrical, and other emerging technologies. For each category, we will examine the operating principles, highlight the advantages and limitations, and explore typical use cases within aerospace systems.

### 2.1. Measurement Based on Optical Principles

**Figure 2:**
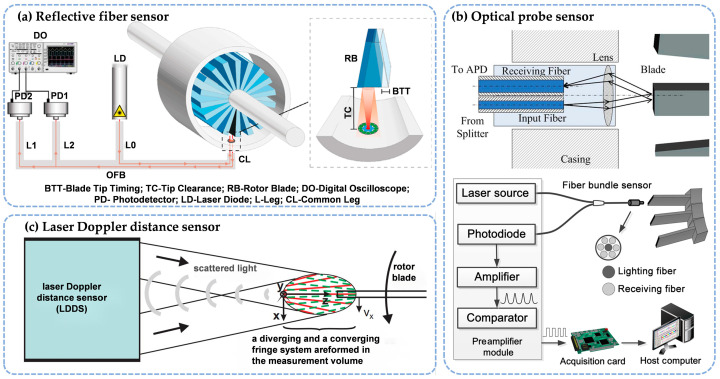
Micro-gap measurement sensors based on optical principles. (**a**) Reflective fiber [19]. (**b**) Optical probe [20]. (**c**) Laser Doppler distance [21].

Optical sensors utilize light, usually transmitted through optical fibers or generated by a laser source, to measure micro-gaps between surfaces. These sensors are highly sensitive, enabling high-resolution measurements without the need for physical contact. This non-contact capability makes optical sensors particularly well-suited for delicate or hard-to-reach components within aerospace systems. Unlike traditional contact-based measurement methods, optical sensors effectively eliminate the risk of friction or damage during measurement, making them ideal for applications that require high precision, especially in scenarios involving small micro-gaps. This section discusses several key optical measurement techniques, including the reflective fiber method, optical probe measurement, laser Doppler distance, and other emerging methods (Figure 2).

#### 2.1.1. Reflective Fiber Method

The basic principle of the reflective fiber method involves transmitting light from a light source to the surface of an object using an optical fiber [22]. As Figure 2a shows, the emitted light is reflected by the blade surface and subsequently captured by receiving fibers. These strategically arranged fibers ensure optimal light collection efficiency across varying gap distances. The collected optical signals are then routed through bifurcated fiber legs to photodetectors, where photoelectric conversion translates light intensity variations into quantifiable electrical signals. Through calibrated intensity-distance mapping, this configuration enables precise determination of micro-gap dimensions with sub-micrometer resolution, particularly effective in confined aerospace environments where physical probe access is limited.

The reflective fiber method has recently gained widespread attention and application in aerospace for micro-gap measurements. For example, Zubia et al. [23] developed a three-pronged fiber reflection sensor capable of simultaneously measuring tip timing and clearance. By mitigating modal noise at the bifurcated end and suppressing speckle noise in the reflected light, they enhanced the system’s accuracy to over 30 µm. This innovation allows for high-precision tip clearance measurements in real engine rotating disks, marking a significant advancement in the field. Similarly, Jia et al. [24] introduced a tip micro-gap measurement system based on intensity-modulated fiber bundles, achieving a measurement accuracy of 25 µm. Coupled with a central signal processing unit and a high-speed data acquisition card, the system demonstrates excellent response characteristics, with promising applications for engine health monitoring and rapid tip micro-gap control. In addition, García et al. [25] validated the performance of a three-pronged fiber sensor for monitoring turntable vibration and later proposed a four-pronged reflection fiber sensor with a wider measurement range (2–4 mm) and improved accuracy (12 µm). These developments underscore the continuous improvement in the accuracy and applicability of fiber-based measurement systems.

Despite variations in design and accuracy, these studies collectively advance the field of micro-gap measurement technology, particularly for aerospace applications where extreme precision is crucial. The reflective fiber method offers several advantages, including high sensitivity, excellent resolution, resistance to electromagnetic interference, and stable performance under harsh conditions. These characteristics make it a valuable tool for micro-gap measurements in challenging environments. However, the method is sensitive to slight tilts in the reflector surface, which can significantly reduce system sensitivity. Additionally, the reflection coefficient of the target object may influence the measurement results, necessitating optimization and compensation in practical applications.

#### 2.1.2. Optical Probe Method

The optical probe measurement method determines micro-gap size based on changes in the path of reflected light [26]. As illustrated in Figure 2b, a typical probe configuration integrates three core components: an input fiber delivering collimated laser emission through a graded-index lens, a reception fiber symmetrically positioned at the lens focal plane to capture retro-reflected signals, and precision optical alignment mechanisms to ensure axial symmetry about the lens centerline. The lens transforms the divergent fiber output into a collimated beam projected onto the target surface (e.g., turbine blade tips), with reflected light being refocused onto the reception fiber through the same optical path. Displacement of the micro-gap alters the beam’s incidence angle, inducing measurable lateral shifts of the focused spot position on the photodetector array. In conventional measurement systems, the reflected light is transmitted using optical fibers to a photoelectric conversion module. Then signals are amplified, compared, and shaped before digitization by a data acquisition card, ultimately enabling real-time extraction of gap parameters through the host computer.

This non-contact approach’s inherent adaptability to high-temperature turbomachinery environments has driven its widespread adoption in next-generation aero-engine health monitoring systems. For instance, Duan’s research group at Tianjin University developed a multimode fiber-coupled laser ranging system. By utilizing multimode optical fibers for light transmission, this system ensures sufficient light power is received by the probe, achieving a resolution of 0.02 mm within a range of 9 mm [20]. Additionally, the same research team introduced a novel technique for measuring rotating blade tip micro-gaps based on blade tip timing and dual-frequency laser phase ranging. By optimizing the RF circuit design, they enhanced the system’s accuracy to 50 µm, offering an innovative solution for high-precision blade tip micro-gap measurement [27]. Further advancements from the group focused on improving the accuracy of tip timing using fiber bundle sensors [28]. Using both hardware and software optimization, they reduced the error caused by variations in the tip micro-gap, validating the method’s effectiveness through experimental testing.

These advancements have not only enhanced the accuracy and stability of the optical probe method but also broadened its practical applicability. Despite its high precision, rapid response, and suitability for dynamic measurements, the optical probe method is still challenged by its high environmental sensitivity and complex assembly requirements. However, ongoing technological innovations and optimizations suggest that this method holds significant promise for applications in fields demanding extremely high precision, such as aircraft engine measurements.

#### 2.1.3. Laser Doppler Distance Method

The laser Doppler positioning method, an emerging optical micro-gap measurement technology, has increasingly become a developed direction in the field of micro-gap measurement [29]. Figure 2c shows the principle this technique relies on. Specifically, the laser Doppler method employs a unique superimposed dual-fringe interference system, where the dual fringes combine both converging and diverging forms, and the spectral separation of the two is achieved using wavelength division multiplexing technology. The integrated laser outputs a beam that is combined using a fiber coupler and transmitted to the probe optical module. When the Doppler-shifted signal light waves scattered by the measured object pass through the measurement area, the receiving optical components capture the corresponding signals, which are then guided by optical fibers to the photodetection unit. Analyzing the Doppler frequency shifts in the two optical channels enables the acquisition of the position and velocity information.

By employing wavelength division multiplexing, two fan-shaped interference fringe systems with opposite gradients can be generated within the same measurement volume. The position of the object can then be accurately determined by analyzing the ratio of the Doppler frequencies of these systems. This approach enables high-precision and high-resolution micro-gap measurements. For example, Neumann et al. [21] proposed an innovative laser Doppler position sensor capable of simultaneously measuring the blade tip micro-gap, blade vibration, and blade speed. This system achieves a spatial resolution of 5 mm, is immune to electromagnetic interference, and is adaptable to blades made from various materials. The method’s high accuracy is not influenced by the speed of the object, and it allows for the synchronous measurement of multiple parameters. Despite its impressive precision, the laser Doppler method faces challenges in practical applications due to its complex system design and high demands for real-time signal processing. These limitations are particularly pronounced in dynamic measurement scenarios, where the need for rapid and accurate data acquisition remains a key obstacle to broader adoption.

#### 2.1.4. Other Optical Measurement Method

Several other advanced optical measurement techniques have emerged in recent years, offering unique advantages in precision, environmental adaptability, and integration flexibility. These methods, including laser interferometric ranging, spectral confocal sensing, and fiber Bragg grating (FBG)-based detection, have demonstrated promising capabilities in non-contact, high-resolution micro-gap measurement-especially under the demanding conditions often encountered in aerospace engineering. This section highlights the working principles, technical progress, and potential applications of these alternative optical methods, which continue to broaden the landscape of micro-gap sensing technologies.

The principle and applied development of laser interferometric ranging technology can be traced back to the research conducted by Kempe’s team in 2003. Utilizing the principle of optical low-coherence interferometry, they constructed a common path interference architecture in which the outer surface of the probe’s optical window served as the reference plane. The blade tip clearance was then determined by analyzing the frequency of the interference signal formed between the reference and measurement beams [30]. Their prototype system integrated a mechanical delay-line interferometer arm and a frequency-shifted reference arm, achieving measurement uncertainty better than 100 μm in a simulated turbine disk experiment. Vakhtin’s team further optimized the sensor head design by employing sapphire fibers and windows to develop a miniaturized, high-temperature-resistant probe (outer diameter 12.7 mm), which achieved a precision of 10 μm within a 0.9 mm measurement range and reduced the measurement time to 20 μs per cycle [31]. This technology exhibits self-calibration capability and maintains robustness under complex thermal and vibrational conditions. However, its resolution and range are theoretically limited by the bandwidth and frequency stability of the light source [32]. Future developments should focus on broadband light source modulation and enhancement of interference fringe signal-to-noise ratio to overcome current performance bottlenecks.

Spectral confocal measurement is a non-contact technique that integrates confocal imaging principles with chromatic dispersion, enabling axial position encoding through wavelength-dependent focusing [33]. By analyzing the reflected wavelength from the target surface and referencing pre-calibrated response curves, precise spatial positioning can be achieved. In recent years, this method has gained traction in high-resolution micro-displacement applications due to its immunity to surface roughness and ambient light interference, as well as its efficiency in eliminating the need for axial scanning. Bi et al. from the Beijing Institute of Aerospace Precision Machinery validated this technique using the confocalDT system from Micro-Epsilon on a rotating blade simulator, achieving a resolution of 60 nm, a range of 1.5 mm, and accuracy better than 1.2 μm [34]. However, limitations in spectrometer sampling speed remain a challenge. Future research should focus on enhancing spectral detection rates to enable broader aerospace applications in precision micro-gap measurement.

Fiber Bragg grating (FBG) sensing technology estimates micro-gap variations by analyzing the wavelength shift caused by strain or temperature changes, based on the linear relationship between these parameters and the grating’s reflective spectrum [35]. Mieloszyk et al. introduced an adaptive wing structural health monitoring (SHM) concept employing FBG sensors integrated with shape memory alloy (SMA) actuators to enable active control of wing deformation [36]. The embedded FBG array was used to monitor surface shape and detect defects in composite skin structures. Due to their immunity to electromagnetic interference, compact size, and lightweight properties, FBG sensors have become ideal tools for aerospace SHM applications. Although currently focused on macrostructural monitoring, the high sensitivity and embedment flexibility of FBG technology offer promising prospects for future use in precision micro-gap measurement scenarios.

Overall, precision micro-gap measurement technologies based on optical principles have been widely applied in the aerospace field. Many commercially available products, such as CREAFORM’s 3D scanner, which is designed to handle a wide range of measurement scenarios, utilize optical principles. These systems employ optical imaging to capture micro-gap images, which are then analyzed using advanced image processing techniques in the post-processing module. The key advantage of this kind of method is that it does not require physical contact with the object, thus avoiding potential damage to the surface while maintaining high measurement accuracy. However, despite these advantages, optical measurement methods are highly susceptible to environmental factors such as light fluctuations and temperature variations. These external influences can impact the accuracy and reliability of measurements. Additionally, while optical methods are effective for many applications, they present challenges when measuring micro-gaps in complex structures or objects with irregular shapes, which may limit their versatility in certain aerospace contexts.

### 2.2. Measurement Based on Electrical Principles

Electrical principles are widely employed in the aerospace industry for fine micro-gap measurement due to their high precision, sensitivity, and adaptability. Unlike optical methods, electrical-based techniques convert physical or mechanical quantities into electrical signals, which are then processed through sensitive elements and circuit signal processing to calculate the micro-gap value. Among these, methods such as capacitive, eddy current, and induction have become well-established and widely utilized in aerospace applications that demand high precision. These technologies are known for their strong resistance to interference, excellent stability, and reliable operation under varying environmental conditions. The following section will provide a concise review and analysis of these electrical-based micro-gap measurement technologies.

#### 2.2.1. Capacitive Method

**Figure 3:**
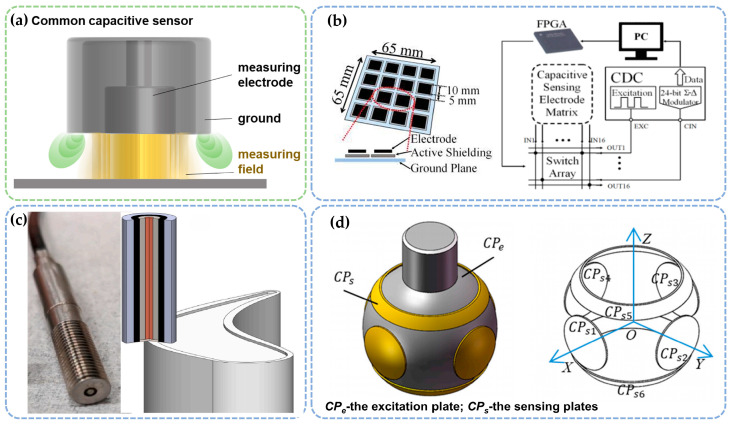
Micro-gap measurement sensors based on the capacitive method. (**a**) Working principle. (**b**–**d**) Typical application scenarios [39,40,43].

The capacitive measurement method is a widely utilized technique for detecting micro-gaps. Its fundamental principle involves monitoring changes in capacitance between the sensor probe and the measured object, which indirectly reflects variations in the object’s micro-gap [37,38]. As shown in Figure 3a, the measuring electrode and ground layer form a parallel plate capacitor configuration. When the micro-gap between the measured object and the measuring electrode changes, the capacitance value exhibits an inverse proportionality to the gap distance. The electric field generated by the measuring electrode penetrates the gap to establish a sensing zone, where gap variations induce distortion in the electric field distribution. This distortion is converted into electrical signals using a high-frequency oscillating circuit. The sensor subsequently demodulates the capacitance variations to achieve non-contact measurement of the micro-gap.

Recent advancements in capacitance-based micro-gap measurement technologies have greatly enhanced their accuracy and applicability (Figure 3b–d). For instance, Xia et al. proposed a tri-mode capacitive proximity sensor comprising a 4×4 electrode array [39] configured to form multiple mutual capacitors for detecting approaching objects through shunting electric fields. The system integrates a four-layer stacked PCB substrate, a field-programmable gate array (FPGA), and a capacitance-to-digital converter (CDC) chipset, with measurement data stored and processed on an external computer. This sensor enables vertical distance assessment, lateral motion tracking, and surface profile recognition, demonstrating promising application potential in the aerospace sector. Wang et al. [40] developed an improved capacitive sensor for detecting micro-gaps in spherical joints. This sensor measures the offset position of the sphere by forming differential capacitance across three capacitor pairs (*CPe* and *CPs*), with the eccentricity of the sphere exhibiting a linear correlation with the displacement when the eccentricity is less than 0.2. However, this method is sensitive to overall measurement errors and presents challenges in practical implementation. In contrast, Satish et al. introduced a capacitive sensor with a larger sensing area (7.6 mm in diameter) [41] that provides smoother signal output for micro-gap ranges from 0.4 to 3 mm, thus improving stability and reliability. Additionally, they proposed a DC bias adjustment circuit based on an RC network [42], enhancing the sensor’s resolution to 2.5 mm, allowing it to detect and adjust low-level capacitance (around 1.25–0.00413 pF). While these developments show promising results under controlled conditions, certain practical challenges remain, particularly in broader industrial applications.

Overall, these studies contribute to the widespread use of the capacitive method in micro-gap measurements. From sensor design to signal processing improvements, they have enhanced measurement accuracy and reliability. However, challenges such as calibration complexity and installation requirements still hinder their more widespread use. Despite this, the capacitive method remains advantageous due to its simple sensor structure, high sensitivity, low power consumption, and robust dynamic response performance.

#### 2.2.2. Eddy Current Method

**Figure 4:**
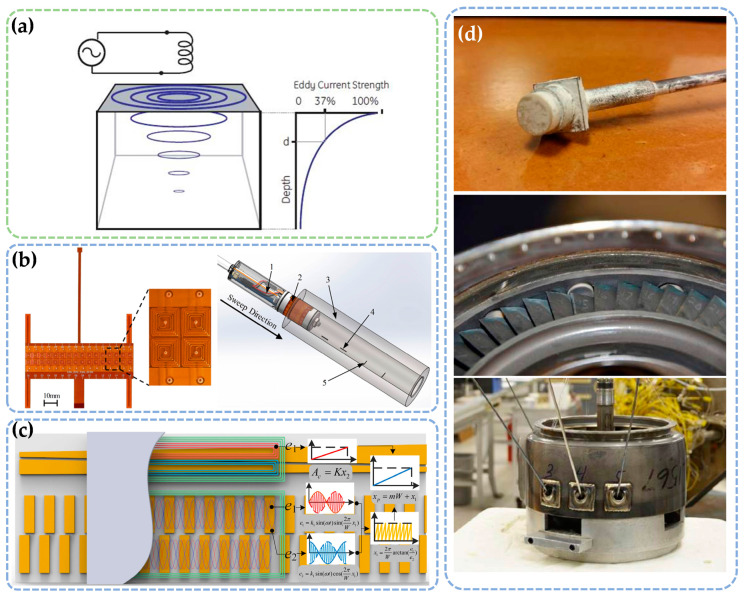
Micro-gap measurement sensors based on the electromagnetic induction method. (**a**) Operating principle. (**b**–**d**) Typical application scenarios [46,47,48].

The eddy current method is a well-established and effective technology for measuring small micro-gaps, particularly in aerospace applications [44,45]. This method leverages the magnetic field changes induced by conductive materials in a magnetic field. As illustrated in Figure 4a, when a sensor coil energized with high-frequency alternating current approaches a conductive object, it generates an alternating magnetic field in the adjacent space. This field induces eddy currents—closed-loop circulating currents—within the metallic object. These eddy currents produce an opposing magnetic field, which alters the coil’s impedance by modifying the original magnetic field. As the gap decreases, the intensified eddy currents induce more pronounced impedance variations, whereas increasing the gap reduces both the eddy currents and impedance changes. A resonant circuit converts these impedance variations into voltage signals, which are subsequently linearized to determine the gap dimensions.

In recent years, the eddy current method has made significant strides in accuracy and versatility (Figure 4b–d). For example, Sun et al. [46] developed a flexible array eddy current sensor capable of detecting a surface micro-gap of approximately 0.3 mm × 8 mm × 0.5 mm in a hollow shaft. Their finite element simulation identified key parameters affecting sensor performance, and optimized configurations improved measurement results. However, the study did not provide sufficient guidance on selecting the optimal parameters for complex real-world applications. V. Sridhar and K. S. Chana improved the design of an eddy current sensor, enabling stable operation at temperatures up to 1400 °C. This sensor, with a compact size of 10 mm, offers high-precision measurements within the micro-gap range of 0–5 mm. Despite its promising performance, the sensor’s vulnerability to friction during operation limits its use in high-friction environments [47]. In contrast, Tong et al. [48] proposed a linear eddy current sensor that achieves high-precision absolute linear position measurements by utilizing both coarse and fine channels. This sensor offers a resolution of 0.25 µm and exhibits excellent linearity across the entire measurement range, making it ideal for applications requiring extremely high precision. These studies highlight the continued advancement of the eddy current method, addressing various challenges such as temperature adaptability, sensor design, and measurement accuracy. However, the method still faces limitations, including dependence on parameter selection, sensitivity to external factors, and potential damage due to friction. Nevertheless, as technology continues to improve, the eddy current method is expected to see broader applications in more complex industrial environments.

The key advantages of this method include its simple structure, compact size, and wide measurement range. However, its application is constrained by requirements for the target material, such as sufficient conductivity and minimum thickness. Additionally, special care must be taken to mitigate the influence of magnetic effects during measurement.

#### 2.2.3. Electromagnetic Induction Method

**Figure 5:**
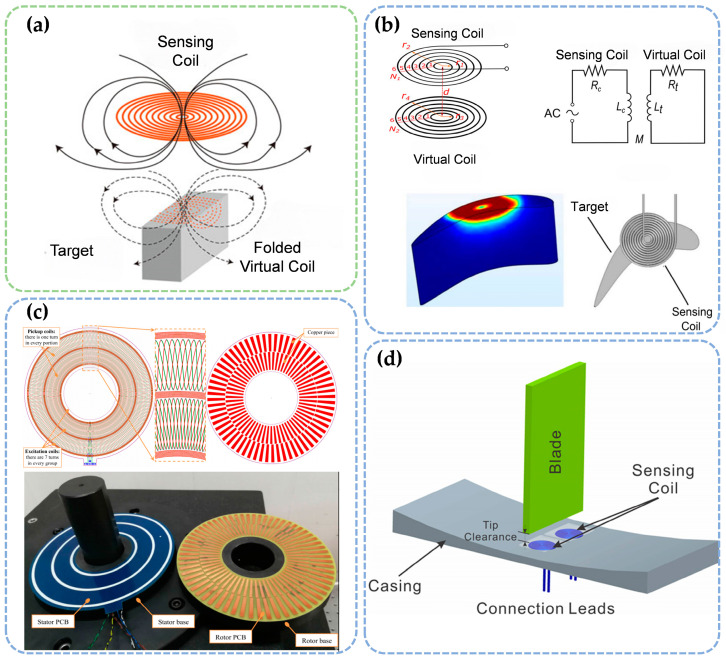
Micro-gap measurement sensors based on the electromagnetic induction method. (**a**) Working principle. (**b**–**d**) Typical application scenarios [49,51,52].

The electromagnetic induction method measures micro-gaps by detecting changes in inductance in a planar spiral coil [49,50]. As shown in Figure 5a, the alternating magnetic field generated by the planar spiral coil induces eddy currents in the nearby metallic target. These eddy currents produce a secondary magnetic field that opposes the original field, thereby altering the overall magnetic field distribution and reducing the effective inductance of the coil. A smaller gap between the coil and the target results in stronger eddy current effects and a more pronounced decrease in inductance. By precisely monitoring these inductance variations and applying appropriate mathematical models, high-resolution, non-contact micro-gap measurements can be reliably achieved.

Recent developments in electromagnetic induction-based micro-gap measurement technologies have led to substantial improvements in both accuracy and application range (Figure 5b–d). For example, Jiao et al. [51] proposed an induction current method that eliminates the need for complex calibration, controlling micro-gap measurement errors within 3.703% by simulating the induced current as a planar coil and performing constant curve corrections. However, this method is restricted to static measurements of a single object, limiting its use in dynamic applications. In contrast, Du et al. [49] developed a multi-channel induction sensor system that employs several micro-planar spiral coils to achieve high-precision measurements with a resolution of 10 µm. This system allows for simultaneous measurement of micro-gap variations at different positions, making it especially suitable for measuring blade tip micro-gaps under 50 µm. While this design significantly improves measurement accuracy and flexibility, challenges related to sensor coordination and data processing remain. Both the simplified calibration approach and the multi-channel sensor system represent important advancements in the application of electromagnetic induction in micro-gap measurement.

Electromagnetic induction-based measurement systems offer several advantages, including long service life, simple structure, and high reliability. However, the performance of these systems may degrade in high-temperature environments as the magnetic properties of permanent magnets weaken. Without additional protection, the working efficiency of these sensors can be compromised.

### 2.3. Measurement Based on Other Principles

In addition to the micro-gap measurement methods based on optical and electrical principles mentioned above, recent years have seen the development of new technologies that employ other physical principles for measurement. Not only do they address the limitations of traditional optical and electrical measurements, but they also provide more precise and reliable results, especially in complex environments. This section focuses on several relatively mature technologies, including ultrasonic measurement, infrared thermal imaging, and discharge probe measurement. Each method is based on a unique principle and exhibits particular strengths in specific applications.

#### 2.3.1. Ultrasonic Method

**Figure 6:**
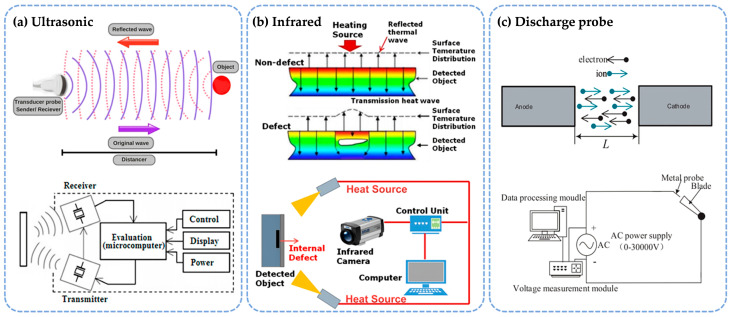
Micro-gap measurement sensors based on other principles. (**a**) Ultrasonic [56]. (**b**) Infrared [57]. (**c**) Discharge probe [58].

The ultrasonic measurement method is a non-destructive testing technique commonly used to detect various defects and measure small micro-gaps based on the propagation characteristics of ultrasonic waves [53,54]. Figure 6a illustrates the fundamental principle and system configuration of ultrasonic distance measurement. The ultrasonic transducer probe integrates both transmitting and receiving functions. The transmitter converts electrical signals into high-frequency ultrasonic waves that propagate through a medium (e.g., air or liquid) to the target surface. The reflected wave, carrying distance information, returns to the probe and is captured by the receiver for conversion into electrical signals. The microcomputer evaluation module within the system precisely calculates the time interval between the transmitted and reflected waves. By combining this interval with the sound velocity in the medium, the target distance is determined. The control and display unit provides real-time measurement outputs, while the power module ensures stable system operation. When a micro-gap is present in the object being measured, the acoustic impedance at the boundary between the micro-gap and the material changes, resulting in phenomena such as reflection and diffraction of sound waves. These alterations in the ultrasonic signal are then analyzed to infer the micro-gap’s size and location. While ultrasonic measurement provides useful information, its accuracy is often limited. For instance, Aliew [55] developed a low-cost ultrasonic sensor module with an accuracy of 0.4 mm. By applying the least squares method, namely, the Vandermonde method, and intelligent filtering algorithms during post-processing, the detection accuracy was significantly enhanced. However, this approach requires real-time monitoring of ambient temperature, which can affect the measurements. The ultrasonic method is particularly suited for measuring complex structures and materials but does require careful consideration of the roughness of the surface of the object being tested.

#### 2.3.2. Infrared Method

Any object with a temperature above absolute zero in nature will radiate infrared energy outward. The infrared thermal imaging measurement method uses this principle [59,60,61]. When there is a micro-gap in the object under test, its temperature field is different from that of the surrounding area. As shown in Figure 6b, a heating source applies controlled thermal excitation to the detected object, propagating thermal energy in the form of heat waves. When internal defects (e.g., micro-gaps) are present, thermal conduction path obstruction occurs, resulting in attenuation of the transmission heat wave intensity and distortion in the propagation characteristics of the reflected thermal wave. An infrared camera captures the surface temperature distribution in real time, identifying defect locations and dimensions by comparing the uniform thermal field in non-defect regions with abnormal temperature gradients in defective areas. The control unit coordinates heating power and data acquisition timing, while a computer performs spatiotemporal analysis of thermal data to achieve non-contact visual inspection. Bo et al. [62] studied the defect detection of carbon fiber-reinforced polymers. Based on infrared lock-in thermal imaging technology, the Fourier transform method and principal component analysis method were combined in the image post-processing process to sort the obtained test sample images and then quantitatively study the position and size information of the defects. When the defect is larger than 6 mm, the error can be controlled within 3%. The infrared thermal imaging measurement method can quickly achieve large-area measurements, but the measurement is often limited to the surface of the object under test, and it is difficult to obtain information in the depth direction.

#### 2.3.3. Discharge Probe Method

The discharge probe measurement method is based on the principle of electric spark discharge [63,64]. Figure 6c illustrates the particle transport mechanisms during gas discharge in electrical discharge machining, where L denotes the inter-electrode spacing. As the applied voltage increases, collisions between charged particles trigger ionization, leading to exponential growth in carrier density. When the voltage reaches a critical threshold, the discharge regime abruptly transitions from Townsend discharge to self-sustained discharge—a phase change phenomenon referred to as gas breakdown. The corresponding critical voltage, which is termed the breakdown voltage, is determined by electrode configuration, gas type, and ambient pressure. In the measurement system, the probe is driven by a stepper motor to move along the object under test. When it moves to the micro-gap, gas discharge occurs between the two, and the probe stops moving. By measuring the distance the probe moves, the size of the micro-gap can be inferred. Yu et al. [55] proposed a novel micro-gap measurement system based on AC discharge. Using experimental analysis of probes with different structures, they selected a flat-head probe with a more stable performance for measuring engine tip clearance. When the micro-gap size is between 0 and 6 mm, the system has high accuracy, and the measurement error is within 0.05 mm. This study optimized the scope of use of the discharge probe method and gave this long-standing method a new lease on life. The discharge probe method has a simple measurement principle. As long as the object to be measured is conductive, reliable measurement can be achieved in a high-temperature and high-pressure environment within a certain accuracy range. However, the mechanical transmission required for the movement of the probe is relatively complex, and there is a risk of collision between the probe and the object to be measured. In comparison, this method is more suitable for experimental research and is often used as a supplementary reference for other methods.

## 3. Comparative Analysis of Applicable Scenarios

As illustrated in Table 2, various micro-gap measurement methods in the aerospace field each possess distinct characteristics, making them better suited for specific applications. For example, contact-based techniques, such as mechanical probes, are advantageous in applications that permit surface contact and demand high absolute accuracy, such as turbine blade tip clearance measurement. In contrast, non-contact optical methods are well-suited for scenarios where surface integrity and measurement resolution are critical, such as detecting micro-gaps between spacecraft structural panels. Electrical sensing methods, while potentially more intrusive, offer robust performance in environments with limited optical access or strong electromagnetic interference, and are commonly used in enclosed or harsh conditions.

Furthermore, ultrasonic and infrared thermographic techniques are particularly effective in the detection of micro-gaps associated with internal defects or material discontinuities, such as delamination, bonding voids, or crack-induced separations. Ultrasonic methods utilize high-frequency sound waves to penetrate materials and are sensitive to internal structural variations, making them suitable for layered composites or subsurface assessments. Infrared thermography, on the other hand, enables non-contact detection based on thermal contrasts and is well-suited for identifying gap-induced thermal anomalies in large structures or during active heating scenarios. These methods complement other measurement techniques by addressing hidden or inaccessible micro-gaps that may compromise structural integrity or reliability in aerospace systems.

These technologies can be employed independently or in combination, depending on the specific requirements for measuring micro-gap in various types and locations. Given the stringent demands for micro-gap measurements in the aerospace sector, precise and reliable measurement techniques are essential to ensuring the safety, performance, and longevity of aircraft and spacecraft.

While Section 3 provides a comparative analysis of various micro-gap measurement techniques and their application-specific advantages, practical aerospace engineering scenarios often demand more than just the selection of a single method. Instead, they require the integration of these methods into comprehensive, high-performance measurement systems tailored to the specific structural and functional needs of aircraft components. Such systems must account for not only measurement accuracy but also adaptability to diverse operating conditions, real-time processing capabilities, and integration into digitalized manufacturing workflows.

To address these requirements, Section 4 delves into representative micro-gap measurement systems that have been developed for critical aerospace assembly tasks. These include systems specifically designed for wing/panel alignment and turbine blade tip clearance, where precise and reliable measurement is essential to ensure performance, safety, and efficiency.

## 4. Micro-Gap Measurement System

Traditional single measurement methods often fall short of meeting the high-precision and high-efficiency requirements demanded by modern aerospace manufacturing and assembly, particularly for large-scale aircraft components. Common methods, such as feeler gauges and human visual inspection, remain prevalent but are subject to inherent deviations due to human subjectivity. These methods also lack efficiency, which limits their ability to align with the growing trend toward digitalization. In cases involving micro-gaps with complex structures, traditional measurement techniques struggle to yield accurate data and pose risks for secondary errors during data storage and implementation. The advent of digital measurement systems has mitigated many of these risks, offering enhanced accuracy and faster feedback and opening new possibilities for intelligent and efficient assembly processes.

For critical micro-gap measurements, such as those involved in the docking of aircraft wings and fuselage [66], as well as between various fuselage components, precision is essential. These micro-gaps have stringent design specifications and significantly impact aircraft performance and overall quality. The following sections introduce several typical micro-gap measurement systems used in aerospace assembly.

### 4.1. Wing/Panel Assembly Micro-Gap Measurement System

The integration of advanced micro-gap measurement technologies into aircraft assembly processes has become increasingly prevalent, with laser measurement technology standing out in this domain. Xu et al. from Beijing University of Aeronautics and Astronautics reported the development of a non-contact measurement method utilizing structured light for the measurement of aircraft skin seams and step differences [67]. This system improves the repeatability and accuracy of micro-gap measurements to within 0.05 mm. However, challenges remain when the measured object—such as small holes or narrow micro-gap—does not allow for effective structured light projection, limiting measurement accuracy. To address this limitation, the dual-line structured light method has been proposed. Li et al. utilized this method to achieve precise measurements of aircraft skin seams, improving measurement accuracy to 0.040 ± 0.002 mm [68]. While this system addresses the errors typically found in traditional methods, it does not provide a solution for measuring micro-gaps between other types of components, highlighting the need for further development in this area.

Long et al. proposed an innovative aircraft skin micro-gap measurement system based on the local density method [69]. This system performs adaptive density adjustment in the region of the skin seam, projecting the resulting 3D data onto a 2D plane to facilitate the extraction of boundaries and key points after detecting the seam. This simplification enhances the computational efficiency of the process. The underlying algorithm employs line and circle fitting techniques, while Gaussian graph clustering is applied to optimize the surface normal and direction vector for curved aircraft skin surfaces. This approach improves the accuracy of surface micro-gap measurements. When compared to actual measurement data, the system demonstrated an average error of just 0.001 mm, highlighting its exceptional precision and minimal deviation. Gong et al. reported a physics-driven digital twin simulation system that provides an effective solution to the problem that current micro-gap measurement relies on manual measuring tools that cannot be followed in real time [70]. Using physical simulation technology, the system can capture the inherent dynamic deformation of the panel and the dynamic deformation during the assembly process, thereby achieving accurate prediction of the micro-gap between the assembled parts. In addition, the system can also calculate and generate a 3D gasket model, optimize the analysis surface through a regression algorithm, and finally export it in a polygonal network format to facilitate the subsequent 3D printing process. The experimental results show that the mean square error between the predicted results and the actual measured value of the system is only 0.008 mm, which significantly improves the assembly efficiency.

**Figure 7:**
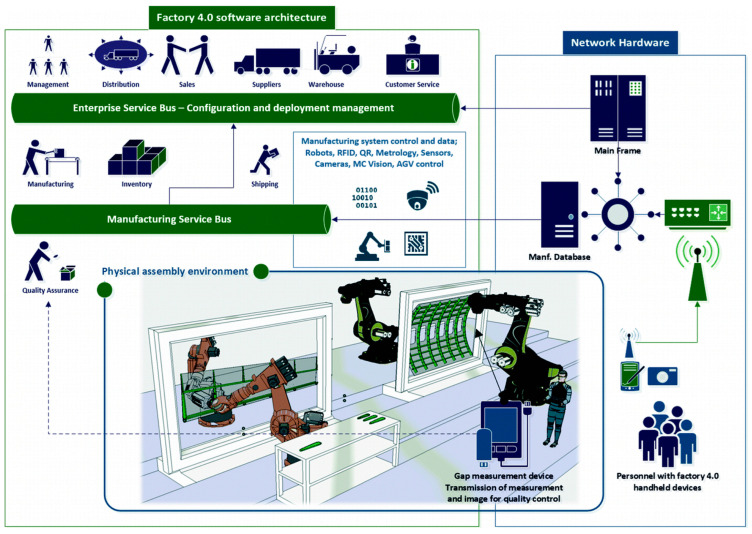
Integration of the handheld machine vision device into the EAS factory 4.0 system architecture [71].

Accurate measurement of the gap between wing skin and rib components is critical to ensuring product quality and structural safety in aerospace manufacturing. Traditional manual methods, such as feeler gauges, are time-consuming, prone to human error, and lack the ability to automatically record and integrate data. While electronic tools like laser scanners and capacitive sensors offer improved precision, their high cost and operational complexity limit their broader deployment. To address these challenges, Crossley and Ratchev introduced a portable, low-cost handheld measurement tool designed using 3D printing and integrated with machine vision [71]. This smart device addresses key challenges in assembly line operations, including the difficulty of camera calibration and lighting consistency, while supporting real-time data transfer within an Industry 4.0 framework. As illustrated in Figure 7, the measurement data collected by the operator is transmitted to the Manufacturing Execution System (MES), which evaluates the data against design tolerances and returns a decision prompt (pass/rework) via an onboard display. In validation experiments simulating aerospace assembly conditions, the system achieved a maximum deviation of 20 μm and a standard deviation of 7 μm for gaps ranging from 0 to 0.7 mm—fully meeting aerospace assembly standards. With future improvements in user interface design and edge compensation for irregular geometries, this system offers significant potential for wider deployment in intelligent and automated assembly environments.

### 4.2. Engine Turbine Blade Tip Micro-Gap Measurement System

Aircraft engines are fundamental components of aviation propulsion systems, and turbine blades play a critical role in large rotating mechanical rotors. Turbine blade tip clearance refers to the micro-gap between high-pressure turbine blades and the engine casing. In this section, we collectively refer to it as the turbine blade tip micro-gap. This micro-gap plays a critical role in determining engine performance, fuel consumption, and overall service life. It is highly sensitive to factors such as uneven airflow and the centrifugal forces generated by the rotor at high speeds. For instance, a 0.105 mm increase in the blade clearance of a high-pressure turbine can reduce engine efficiency by 0.5%, while fuel consumption can increase by 0.25% [72]. Given the complex range of factors affecting turbine blade tip clearance, accurate and reliable measurements are essential to support performance analysis, optimization, and subsequent improvements.

Han et al. proposed a dynamic, high-speed measurement system for engine blade tip clearance based on an optical comb and time-stretched dispersive Fourier transform technology [73]. This system has, to a certain extent, broken the limitations of traditional optical measurement equipment in terms of measurement speed, achieving a measurement error of less than 1 μm and a measurement speed of up to 17.6 MHz. In addition, the system reduces the requirements for data acquisition card sampling rate and detector bandwidth, providing solid technical support for engine health monitoring. On the other hand, He et al. developed a portable turbine blade tip clearance measurement system based on a high-speed camera, providing an effective solution for real-time blade tip clearance measurement in harsh working environments [74]. By constructing an image processing algorithm and analyzing video or image sequences in real-time, the distance between the blade tip clearance and the turbine can be accurately calculated. Compared with other direct measurement methods, this system has significant advantages in flexibility and hardware maintenance costs.

Currently, a variety of technologies are employed to measure blade tip clearance, including discharge probe methods, capacitive measurement techniques, and microwave-based methods. For example, the Rotatip measurement system by the British company ROTADATA, which utilizes the discharge probe method, can achieve a resolution of 0.01 mm at a working speed of 5000 rpm, with repeatability controlled within 0.05 mm. The capacitive measurement method, known for its simplicity and efficiency, is widely used in various products, such as the AS-5000 sensor developed by Milliren Technologies, which offers an accuracy of up to 0.0025 mm. Additionally, the microwave sensor developed by the Beijing Great Wall Metrology Institute, based on the microstrip patch principle, operates in a 24 GHz working environment, offering a measurement sensitivity of 0.5°/mm within a 0.1–6 mm range.

**Figure 8:**
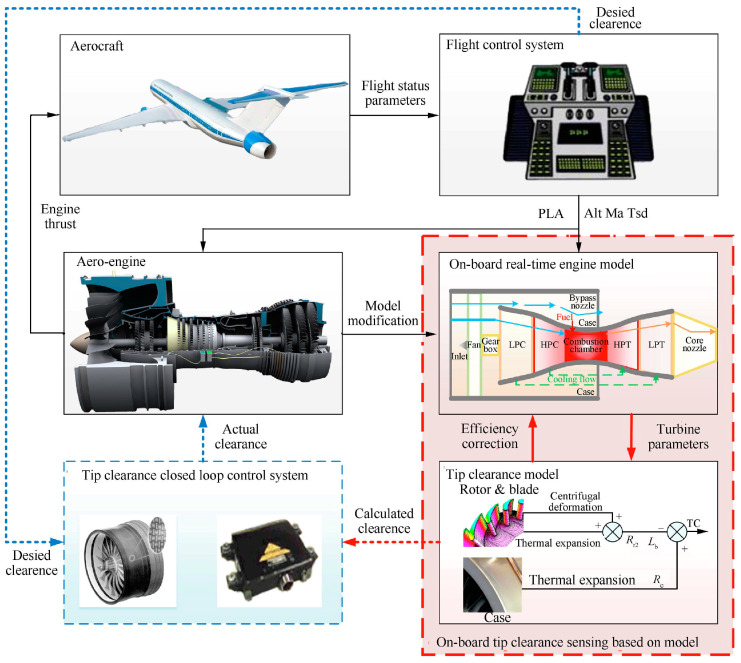
Model-based onboard perception method for tip clearance and its application scenarios [75].

In addition to traditional sensor-based approaches, recent advances have introduced physics-informed modelling techniques for real-time prediction of turbine blade tip clearance under extreme operating conditions. Due to the limitations of physical sensors such as fiber optic and eddy current probes—namely complex installation, high cost, and limited reliability in high-temperature, high-pressure, and high-vibration environments—Sheng et al. proposed a model-based method that dynamically simulates thermal and centrifugal deformation of turbine components [75]. As illustrated in Figure 8, by integrating predicted clearance values into the engine control system, this method improves turbine efficiency and reduces fuel consumption. The model, tightly coupled with the engine’s dynamic system and embedded within the Engine Health Management System (EHMS), was validated using real-time simulation. The results showed tip clearance varying from 1.0 mm during takeoff to 0.3 mm during cruise, causing efficiency fluctuations up to 3.28%. With an average computation time of 0.34 ms, it meets the real-time requirements of control systems (20 ms step size), offering a robust and cost-effective solution for active clearance control in next-generation intelligent aero engines.

Although recent advancements in micro-gap measurement systems have significantly improved the precision and efficiency of critical aerospace assembly tasks, several limitations remain. Many existing solutions are tailored to specific applications, with performance often constrained by environmental factors, structural complexity, or a lack of real-time adaptability. Furthermore, as aircraft design and manufacturing become increasingly digital and automated, the expectations for integrated, intelligent measurement capabilities continue to grow.

In light of these evolving demands, the following section explores the future development directions and challenges in the field of precision micro-gap measurement. It highlights key areas where continued innovation is essential to meet the performance, robustness, and integration requirements of next-generation aerospace systems.

## 5. Future Development Directions and Challenges

As aerospace technology continues to advance, the need for precise micro-gap measurement becomes increasingly critical across all stages of development, from design and manufacturing to operational use. While current measurement technologies have made significant progress, several key challenges remain, particularly in achieving higher accuracy, greater environmental robustness, and seamless system integration under extreme conditions. This section outlines the main challenges in the field of precision micro-gap measurement and explores potential future directions aimed at addressing these challenges. Specifically, it examines innovations in sensor technologies, system integration, and the evolving demands of digital and automated manufacturing, all of which will shape the future of micro-gap measurement in the aerospace sector.

- Enhancing measurement accuracy
  As modern aircraft structures grow in complexity and performance requirements become more stringent, the need for ultra-precise control of micro-gaps during both manufacturing and in-service inspection is becoming more critical. Improving measurement accuracy is essential for the reliable acquisition, analysis, and optimization of micro-gap data. Key research efforts are focused on achieving high-resolution identification of transitions from regular to irregular geometries, which is vital for maintaining component performance, aerodynamic stability, and safety standards.
- Minimizing environmental impact
  Aerospace systems operate in harsh and often unpredictable environments, including high temperatures, pressures, vibrations, and dynamic loading conditions. Stable micro-gap measurement under such challenging conditions is essential for ensuring the safe operation and longevity of aerospace components. Future measurement technologies must focus on minimizing environmental sensitivity, simplifying measurement structures, and enhancing adaptability to these harsh conditions. Robust calibration and error compensation models will also be essential to maintaining consistency across varied applications.
- AI-Driven Digital Inspection
  The evolution of smart manufacturing and digital engineering has reshaped how measurement systems are integrated into aircraft development workflows. The fusion of conventional gap measurement methods with real-time data analytics, artificial intelligence (AI), and digital twin frameworks offers tremendous potential for adaptive, high-efficiency gap control. AI-enabled systems can perform intelligent noise filtering, feature extraction, and fault prediction, while digital twins allow for virtual simulation and optimization of gap dynamics across the entire lifecycle of aerospace components. These technologies are expected to enable real-time feedback loops between virtual models and physical measurements, dramatically enhancing precision and responsiveness in complex production environments.
- Scalability and standardization for large-scale aerospace systems
  One of the most pressing future challenges lies in ensuring that advanced gap measurement technologies are scalable and deployable across large-scale aerospace systems. This includes the development of lightweight, portable inspection equipment capable of high-accuracy measurements in confined or hard-to-reach areas, such as fuselage joints or engine interiors. Furthermore, standardizing these technologies across robotic platforms, unmanned systems, and modular assembly lines is crucial to enabling interoperability, minimizing setup time, and reducing integration costs. Research on universal sensor-mounting frameworks, plug-and-play communication protocols, and cross-platform software compatibility will be essential to achieve widespread adoption.

The next generation of micro-gap measurement systems in aerospace engineering must be not only more accurate and environmentally resilient but also digitally integrated, intelligent, and scalable. Addressing these interdisciplinary challenges will require close collaboration between metrology experts, AI researchers, systems engineers, and aerospace manufacturers. With continued advancements, these technologies have the potential to transform the precision and efficiency of future aerospace design and operation.

## 6. Conclusions

We have summarized the principles, advantages, limitations, and practical applications of several micro-gap measurement methods that are widely employed in aerospace engineering. While significant advancements have been made, challenges persist, particularly in applying these technologies with high precision in dynamic and harsh aerospace environments. As the aerospace sector evolves, the demand for greater measurement accuracy, extended detection ranges, and enhanced resilience to environmental factors continues to increase. Moving forward, the key challenges for micro-gap measurement technology will lie in achieving high precision in complex, fluctuating environments; improving the adaptability and stability of measurement methods; and enabling real-time, high-throughput measurements. These advancements are crucial for meeting the growing demands of aerospace design, manufacturing, and operational processes while contributing to the overall optimization of aircraft performance and safety.

## Figures and Tables

**Table 1:** Micro-gap requirements for the assembly or tip of various aircraft components.

Micro-Gap Position	Micro-Gap Value Requirement (mm)
High-pressure turbine blade tip [11]	0.199–0.625
Aircraft skin assembly [12,13]	0.15–0.4
Cabin door stop block assembly [14]	0–0.5
Wing assembly [9,15,16]	1–1.5
Turbine blade assembly [17,18]	0.2–3

**Table 2:** Comparisons of different types of micro-gap measurement methods.

Type	Detection Method	Measurement Performance	Advantages	Disadvantages	Applicable Scenario	Ref.
Optical	Reflective fiber optic	Accuracy ≤30 µm	High sensitivity, high resolution, anti-electromagnetic interference, stable performance	High requirements for the measured object and environment	Engine blade tips	[23]
		Accuracy 25 µm				[24]
		Range 2–4 mm; Accuracy 12 µm				[19]
	Optical probe	Accuracy 50 µm	High measurement accuracy, fast response frequency, suitable for dynamic measurement	High environmental requirements and complex assembly process	Wing assembly	[65]
		Resolution 0.02 mm			Engine blade tips	[20]
	Laser doppler positioning	Resolution 5 mm	High accuracy, insensitive to object movement speed, measure multiple parameters simultaneously	Complex structure and high demands for subsequent signal processing	Engine blade tips	[21]
Electrical	Capacitive	Resolution 2.5 mm	High sensitivity, low power, good dynamic response performance	Complex device installation and calibration	Spherical joints	[42]
		Range 0.4–3 mm			Engine blade tips	[47]
	Eddy current	Range 0–5 mm	Simple structure, small size, wide measurement range	High requirements for the measured object	Concentric axis	[48]
		Resolution 0.25 µm			Engine blade tips	[49]
	Electromagnetic induction	Range 0.5–3.6 mm	Long lifespan, simple structure, high reliability	Difficult to use in high-temperature environments	Narrow and irregular targets,	[51]
		Resolution 10 µm			transmission wheel	[56]
Other measurement methods	Ultrasonic	Range 0–6 mm Accuracy 0.4 mm	High sensitivity, high measurement efficiency, simple equipment	High requirements for the surface roughness of the measured object	Thin-walled tubes and pipes	[58]
	Infrared thermographic	Range 2–10 mm	Fast measurement speed, large-range measurements	Difficult to detect in the depth direction	Material defects	[62]
	Discharge probe	Range 0–6 mm	High-temperature resistance, low requirements for the shape of the measured object	Accuracy is heavily influenced by the environment	Engine blade tips	[55]

## Data Availability

Not applicable.
